# Malaria incidence and prevalence on Pemba Island before the onset of the successful control intervention on the Zanzibar Archipelago

**DOI:** 10.1186/1475-2875-9-32

**Published:** 2010-01-25

**Authors:** Thomas Jaenisch, David J Sullivan, Arup Dutta, Saikat Deb, Mahdi Ramsan, Mashavu K Othman, Roger Gaczkowski, James Tielsch, Sunil Sazawal

**Affiliations:** 1 Department of International Health, Bloomberg School of Public Health, Johns Hopkins University, Baltimore, USA; 2 Public Health Laboratory - IdC, Pemba, Zanzibar, Tanzania; 3 Institute of Public Health, Heidelberg University Hospital, Heidelberg, Germany; 4 Zanzibar Malaria Control Programme, Zanzibar, Tanzania; 5 Department of Microbiology and Immunology, Bloomberg School of Public Health, Johns Hopkins University, Baltimore, USA

## Abstract

**Background:**

Malaria incidence has been reported to decrease substantially in parts of sub-Saharan Africa, including the Zanzibar Archipelago in East Africa. A cohort study with an intensive follow-up on Pemba Island just before the onset of the highly successful malaria control intervention was conducted. The reported estimates of parasite prevalence and incidence can serve as a robust baseline to evaluate the effect size of the successful interventions and the potential contribution of quality controls and other factors associated with research studies in the decreased estimate of transmission.

**Methods:**

In a rural clinic, two successive cohorts of 537 children total aged 2-23 months were followed for six months each with an intensive visitation schedule of bi-weekly follow-up. Robust estimates of incidence and prevalence according to four different malaria definitions were obtained.

**Results:**

Malaria incidence and prevalence placed Pemba Island in a hyperendemic rather than holoendemic setting for the years 2003-2005. Overall parasite prevalence was estimated to be 39% - with monthly estimates varying between 30% and 50%. Incidence of malaria varied between 2.3 and 3.8 malaria episodes per year based on a diagnosis of fever and various microscopy-based parasite thresholds and between 4.8 and 5.7 based on a diagnosis of fever and 100 parasites/microliter analogous to detection by rapid diagnostic tests. Both parasite densities and malaria incidence increased with age and rainy season. Malaria incidence also varied substantially between the individual villages within the study area.

**Conclusions:**

Pemba Island was previously considered holo-endemic for Malaria. The data suggest that the transmission situation on Pemba Island was significantly lower in 2003-2005 suggesting a hyper-endemic or meso-endemic transmission environment. The figures were obtained just before the onset of the highly successful malaria control intervention by impregnated bed nets and IRS on the Zanzibar Archipelago and provide robust estimates of the malaria transmission situation prior to the control programme. Together with other published data, the results suggest that malaria transmission had started to decrease before the onset of the control programme. The local heterogeneity in malaria incidence highlights the importance of a micro-epidemiological approach in the context of malaria control and elimination.

## Background

Malaria incidence has reportedly decreased substantially in parts of sub-Saharan Africa, including the Zanzibar Archipelago in East Africa. A number of reasons have been proposed to explain this positive trend - among them successful control interventions, more specific diagnostic capabilities, and demographic patterns including better access to care. However, the extent of the decrease is still somewhat surprising and leaves room for speculation if the full range of explanations is being properly evaluated.

The results are reported of a cohort study that was carried out on Pemba Island, which is located north of Zanzibar Island and is part of the Zanzibar Archipelago. The island is densely populated with approximately 300,000 people in an area roughly 70 km long and 30 km wide. Malaria had almost been eradicated on the island in the 1950's. By 1979, 11 years after the suspension of the control programme, malaria had re-established itself on the island [1]. At the time of this study (2003-2005), Pemba Island was considered holo-endemic for malaria [2,3], with perennial transmission peaking shortly after the two rainy seasons. *Plasmodium falciparum *malaria is the leading cause of morbidity and mortality in children on the island of Pemba [4,5]. The study was performed just before the start of the highly successful transmission reduction programme, that used insecticide-treated bed nets (ITN) and indoor residual spraying (IRS) [6]. Artesunate combination therapy (ACT) was introduced in September 2003 according to the Zanzibar Ministry of Health guidelines - however, ACT was not widely available in government health facilities on Pemba Island at the time of the study.

Incidence and prevalence estimates of the pre-intervention period are essential to evaluate the impact of the current programme. Robust estimates of parasitaemia prevalence including quality control of microscopy and robust estimates of incidence according to three laboratory-based malaria definitions and the presumptive IMCI definition were calculated. In addition to seasonal variation of parasite prevalence and malaria incidence using four different definitions, the local heterogeneity of incidence in the micro-epidemiological context of central Pemba Island was described.

## Methods

### Study design

The study took place between December 2003 and January 2005 in three adjacent administrative units ("shehias") on Pemba Island, Zanzibar, Tanzania. The shehias of Kiungoni, Pandani and Finya are located in Wete and Michweni District, about halfway between Chake Chake and Wete Towns. Kiungoni, Pandani and Finya are comprised of 17 sub-villages distributed over an area of around 65 square kilometers. The area has a rural character with fishing, farming and clove production as main income generating sources.

The design was a prospective cohort with bi-weekly active follow-up over a period of 12 months. Two cohorts of 298 and 253 children each, aged 1-23 months at enrollment, were recruited and followed for six months each over the period of one year total. The children enrolled represented 80-90% of the total children per village in the age range of 3-15 months.

Informed consent was sought with the village committee first and after that with individual caregivers. Written informed consent was acquired from every guardian before a child was enrolled into the study. None of the guardians of a child declined consent at enrollment. Ethical clearance was obtained from the Johns Hopkins Bloomberg School of Public Health Institutional Review Board and the Zanzibar Health Research Council.

The study was nested within a larger zinc/iron supplementation trial with a two-by-two-factorial design that was recently completed on Pemba Island, Tanzania [3]. All necessary infrastructure for field visits and specimen collection were already present. At the time when this malaria study started, the zinc/iron trial had been reviewed by the Data Safety and Monitoring Board (DSMB) and the iron component was discontinued. Thus, for the duration of this study, supplementation was either zinc or placebo (which was vitamin A) [7].

### Study population

537 children aged 1-23 months at enrollment were included in the analysis. Table 1 shows the basic demographics of those 537 children. Children in the second cohort came from families with a higher family-income on average (p < 0.001). Also bed net use at enrollment was more prevalent in the second cohort (p < 0.001). Age and gender were equally distributed between the two cohorts.

**Table 1:** Demographic characteristics of 2 cohorts of children on Pemba Island, Zanzibar

	First cohort (N = 291)		Second cohort (N = 246)	
	
	No.	%	No.	%
Age at enrolment				

1-6 m	109	37	97	39

7-12 m	130	45	75	31

> 13 m	52	18	74	30

Gender				

Male	142	49	111	45

Female	149	51	135	55

Household income ($/month)				

< 10	132	45	33	13

Oct-40	147	51	179	73

> 40	12	4	34	14

Bednet use by child				

No	191	66	111	45

Yes	67	23	129	48

Don't know	33	11	16	7

Study period	December 15, 2003-June 15, 2004		August 15, 2004-January 15, 2005	

Children were censored from the study when their caregivers either actively refused further participation or when the children missed four consecutive visits that were scheduled every two weeks. Altogether 67 children (12.2%) were lost to follow-up - most of them due to refusals (N = 37) or outmigration (N = 15). Data on these children were used for analysis up to the point of censorship. 13 children had to be censored completely from the analysis because they only presented for the enrollment visit. One child was removed from the analysis because of ineligible age at enrollment (49 months). Two children were referred for further medical treatment by the study team and eight children died during the observation period.

### Data and sample collection

At baseline, a clinical examination and a structured questionnaire were administered and a capillary blood specimen was taken. After the baseline examination, every two weeks a follow-up clinical examination and a capillary blood specimen for malaria smear, full blood count, including haemoglobin, and a PCR-filter paper for later genotyping of the parasites, were conducted.

A rural clinic was held every day except weekends. Mothers were informed about the results of the blood smear, haemoglobin and white blood cell counts from the previous day and given malaria treatment if appropriate for their children according to WHO recommendations and guidelines of the Ministry of Health (MOH) in Zanzibar. Mothers/caretakers of the children were given a scheduled date of return approximately two weeks after their last visit each time they attended the clinic even in the absence of any health problem. The mothers were encouraged, however, to attend the clinic any day of their choice in case of a health problem with their child. The clinic was open five days a week for children up to five years of age, so that elder siblings of the children enrolled in the study were eligible for free treatment. Free treatment was also given for non-malarial illnesses like diarrhoea, pneumonia and ARI, skin infections (both bacterial and fungal) or otitis media, which could be treated in a peripheral health care setting. If hospitalization was necessary, the child was given free transport to the main hospital of the island on the same day. If a child was investigated for a health problem at a given day, the next visit was scheduled two weeks from that date of investigation.

Blood slides for malaria parasites were prepared according to standard protocols and read against 200 white blood cells [3,8]. Standard quality control procedures included microscopist reading blinded his or her own slides from the previous day as well as those of other microscopists. Double data entry occurred every day in two shifts in a data entry facility at the Pemba Public Health Laboratory (PHL), together with data from the "umbrella" zinc-iron-trial, in which the study was nested. Quality control measures and internal checks for data entry were implemented. Data were analyzed using STATA 9.2 (StataCorp, College Station, TX, USA).

### Malaria definitions

Three laboratory-supported definitions of a clinical malaria episode and the presumptive IMCI definition of clinical malaria were used:

1. A child presenting with 5,000 parasites per microliter plus history of fever (last 48 h) or fever (≥ 37.5°C) at presentation [9].

2. A child presenting with either a) documented fever (≥ 37.5°C) and a parasite count of more than 1,000 per microliter; b) history of fever (last 48 hours) and a parasite count of more than 3,000 per microliter; or c) a parasite count of more than 8,000 per microliter. This definition was developed and used in the zinc/iron trial [3,7], in which this study was nested.

3. A child presenting with ≥ 100 parasites per microliter plus history of fever (last 48 h) or fever (≥ 37.5°C) at presentation. This definition represents the performance of most malaria rapid diagnostic tests (RDT) or "malaria dipstick test". It is also very close to a definition occasionally used in the literature of 'any parasitaemia plus history of fever (last 48 h) or fever (≥ 37.5°C) at presentation'.

4. A child presenting with history of fever (last 48 h) or fever (≥ 37.5°C) at presentation and no sign of invasive bacterial disease (e.g. bacterial pneumonia) by clinician's judgement (IMCI definition).

The monthly malaria incidence rate was calculated using a person-time approach. For a conservative estimate missed days (defined as any days exceeding the retrospective assessment of 14 days prior to a visit to the clinic) were subtracted from the denominator as well as days not under risk if a child was diagnosed with a malaria episode (where the following seven days were subtracted from the denominator). The incidence rate was calculated just based on the total follow up time per child (defined as the period between first and last visit). Both estimates give a plausible range for the true malaria incidence rate per malaria definition.

## Results

Between 10 and 15 visits were recorded per child (mean 12.6; SD 2.6) and compliance was very good. Eight children died with unknown cause (a mortality rate of 3% per year). All deaths were followed up with verbal autopsies within four weeks of the death. In one case, the child died immediately after enrolment indicating that the illness was already in progress. In the majority of the other cases a probable diagnosis leading to death could be made. An acute fever episode with or without convulsions was the most frequent clinical picture leading to death, which results in malaria and/or pneumonia being the most likely diagnoses. Measles was included in the cause of two deaths. A total of 6,125 visits were counted over total follow-up time. At every visit, except 14 with missing data (where a blood slide could not be obtained because of technical reasons or objection from the caretakers), a blood-slide for malaria parasites was taken and analysed according to standard procedures [3]. Altogether 2,331 out of 6,111 blood slides (38.1%) over the whole period of about one year (December 2003 - January 2005) were positive for asexual stages of *P. falciparum*. Documented fever (temperature ≥ 37.5°C) was present in 26% of the parasite-positive visits and in 11% of the parasite-negative visits. Fever either by history or documented was present in 48% of the parasite-positive visits and in 28% of the parasite-negative visits. Asymptomatic parasitaemia was defined as any parasite load (by microscopy) greater than zero without fever (≥ 37.5°C) or negative history of fever in the prior 48 hours. Asymptomatic parasitaemia was confirmed in 19.1% of all visits and in 5.8% and 2.5% with parasite densities above 1,000 and 5,000 parasites per microliter respectively. Only 39% of the children in this study were reported to sleep under a bed net at the time of enrollment (see Table 1).

**Figure 1:**
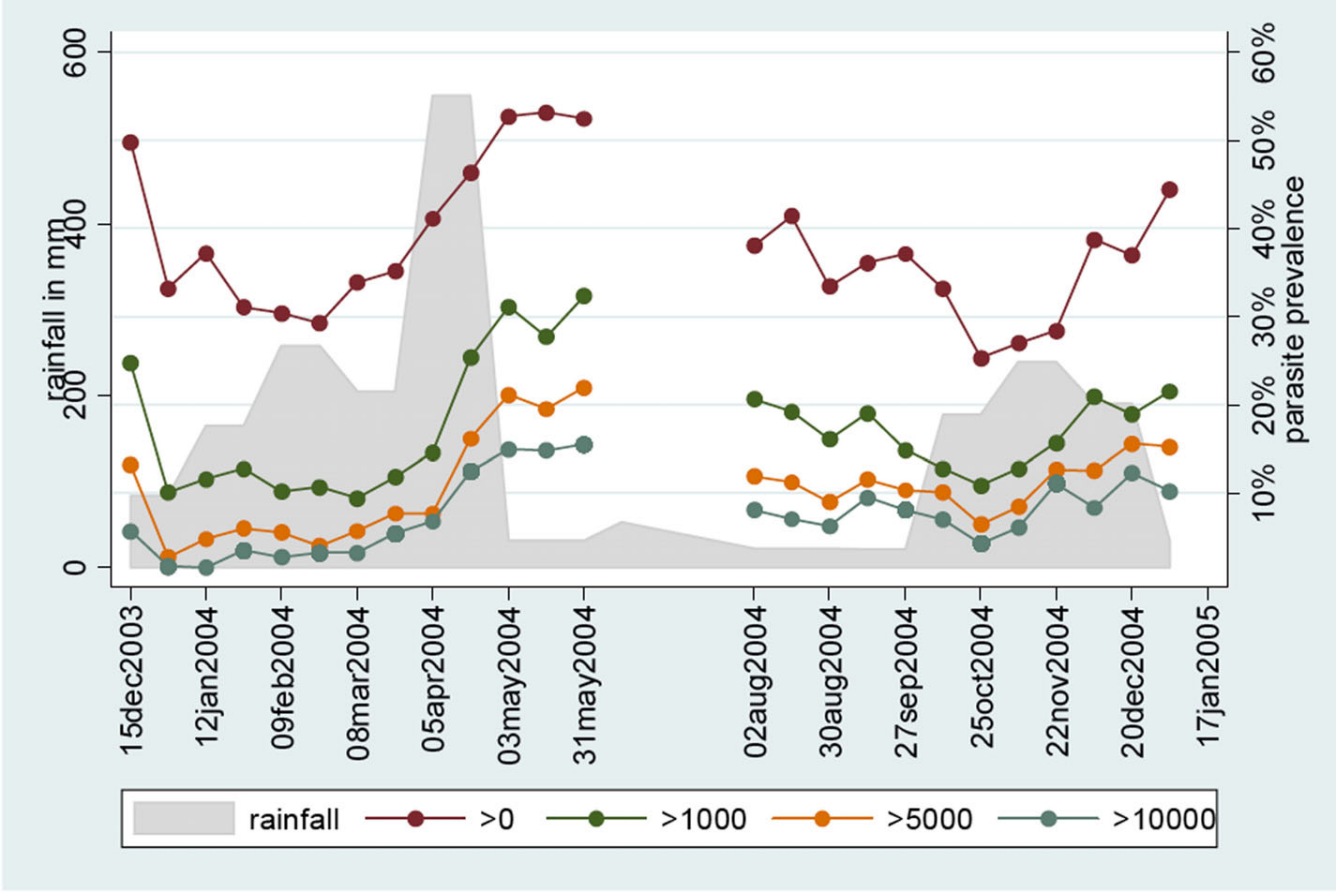
**Rainfall and parasite prevalence by microscopic densities per microliter per 2-week period in two cohorts totaling 537 children on Pemba Island**. Any microscope positive bloodfilm (red circle); greater than 1,000 parasites/microliter (green circle); greater than 5,000 parasites/microliter (orange circle); and greater than 10,000 parasites/microliter (grey circle).

Parasite prevalence per two-week period ranged between 30-50%. Absolute parasite prevalence as well as relative parasite densities were highest during and shortly after the rainy season in April/May and November/December (see Figure 1).

**Figure 2:**
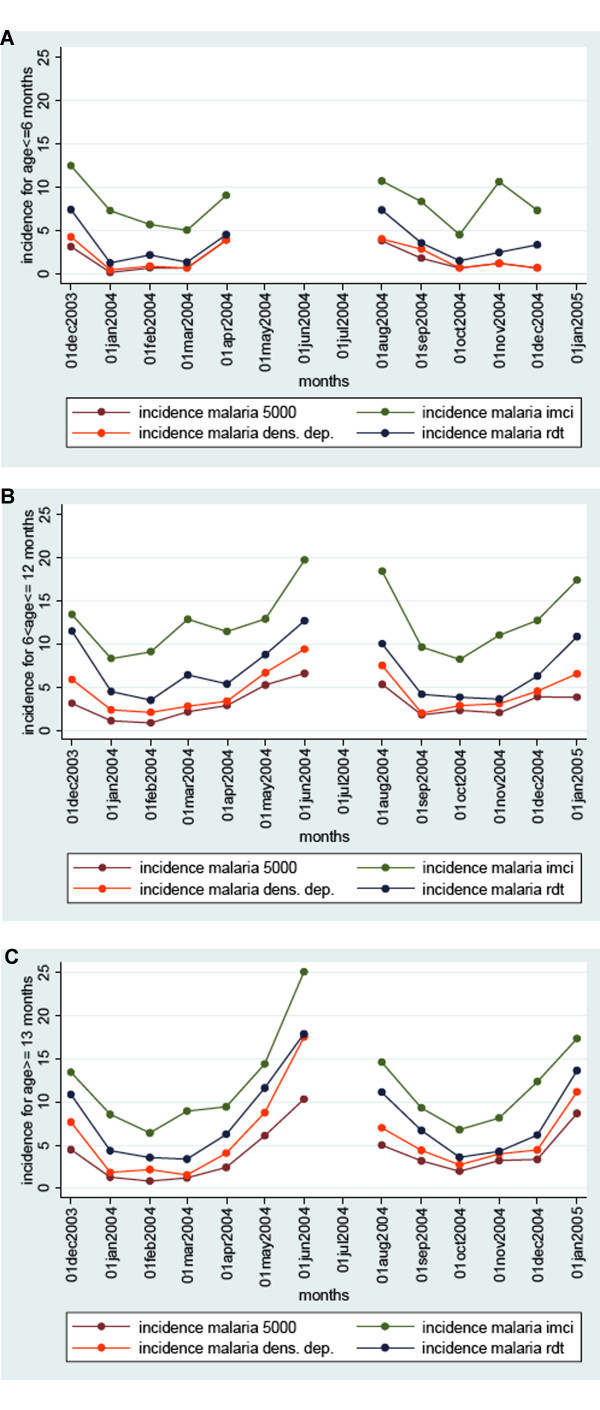
**Yearly incidence per month per three age groups in a cohort of 537 children on Pemba Island by 4 malaria definitions**. Any fever (green); fever and confirmed microscopy with greater than 100 parasites/microliter (blue); documented fever and greater than 1,000 parasites or any fever and greater than 3,000 parasites or greater than 8,000 parasites (orange); and any fever and parasites greater than 5,000/microliter (brown). Age at start of study A) ≤ 6 months; B) 7-12 months; and C) 13-22 months. Cells with less than 500 days of risk in the denominator were omitted from the analysis.

For malaria incidence, three laboratory-based malaria definitions (two involving microscopy and one involving a RDT) were compared to the presumptive IMCI definition. By the rather stringent definition of malaria with a parasite threshold of 5,000 per microliter, malaria incidence is estimated to lie between 2.3 (based on total follow up) and 2.6 malaria episodes per child per year in Figure 2. Using the malaria definition adapted from recent research on the island (varying parasite threshold level according to ascertainment of fever) incidence is estimated between 3.2 and 3.8 episodes per child per year. If a definition with a detection threshold of 100 parasites per microliter is used, between 4.8 and 5.7 episodes per child per year are estimated. This definition is similar to detection thresholds of currently used RDTs. By the ICMI definition of documented fever or history of fever without signs of invasive bacterial disease between 8.7 to 10.2 episodes per child per year are estimated.

**Figure 3:**
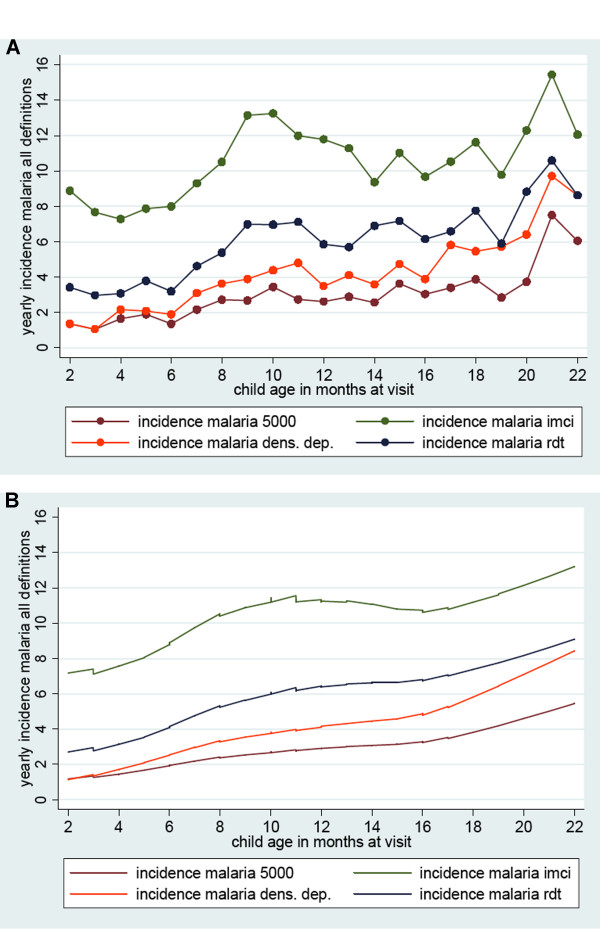
**Yearly incidence by age in months according to 4 different definitions of malaria in two cohorts of 537 children total**. Any fever (green); fever and confirmed microscopy with greater than 100 parasites/microliter (blue); documented fever and greater than 1,000 parasites or any fever and greater than 3,000 parasites or greater than 8,000 parasites (orange); and any fever and parasites greater than 5,000/microliter (brown). A) Estimate per month of age; B) Smoothing by lowess regression. The 95% confidence level was within +/- 40% of the value plotted.

If incidence is calculated separately in three age groups over the study period (1-6 months; 7-12 months; 13-24 months) a seasonal effect is present as with parasite prevalence (see Figure 3). The IMCI definition gives much higher estimates of incidence in the two younger age groups (during the first year of life), whereas in the children from 13-24 months the difference between the IMCI definition and the other three definitions is less pronounced. Yearly incidence estimates increase with age at different rates, but with a similar pattern, as presented in Figure 3. By the end of the first year, they seem to reach a plateau or increase at a slower rate.

**Figure 4:**
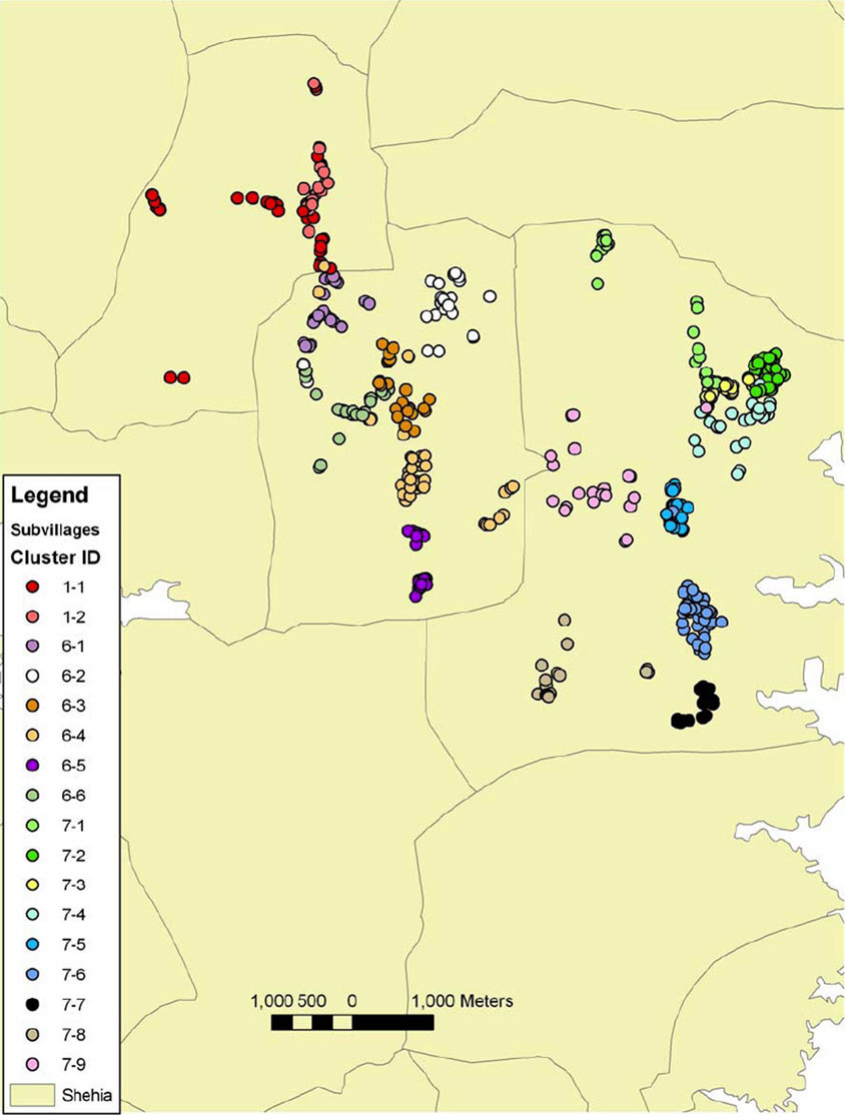
**Map of the area on Pemba Island with geographical locations of 537 children in 17 sub-villages**.

**Figure 5:**
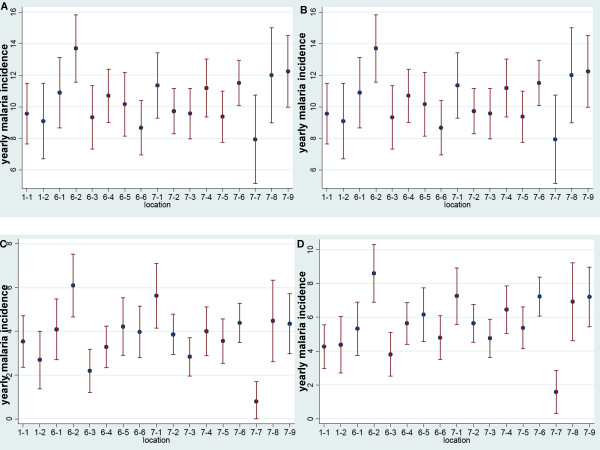
**Local heterogeneity in yearly malaria incidence: incidence is presented per subvillage (N = 17) including 95% confidence intervals**. A) malaria 5,000 definition; B) IMCI definition; C) density-dependant definition; D) RDT definition.

Yearly malaria incidence varied substantially between the 17 (sub)villages within the study area mapped in Figure 4 (each with on average 31 children; range 14-53). Figure 5 shows the variation in incidence (including confidence intervals) for the four definitions. Incidence measured by the strict 5,000 definition varied between 0.5 episodes per year and five episodes per year depending on location. The incidence by the IMCI definition not only provides the highest average, but also the biggest variations. The density dependant definition is comparable to the strict 5,000 definition, with some more variability between the villages. The RDT based definition was approximately 60% of the IMCI definition. Two villages were well outside the range of most other villages throughout all four definitions (village 6.2 with high incidence and 7.7. with rather low incidence).

## Discussion

Estimates of malaria incidence estimates are highly dependant on the definition used. By using four different definitions to provide a plausible range of incidence in order to make comparisons across sites was attempted. In cases where the absolute number of episodes per year is difficult to compare across sites, the age pattern of incidence can still be a good point for comparison. Incidence rates in holo-endemic regions as in the mainland of Tanzania are reported to peak towards the end of infancy and then decrease [10]. A pattern was described consistent with less intense transmission, where an increase in the first year of life is observed and a plateau phase or slower increase during the second year of life (Figures 3a and 3b).

Pemba Island was previously defined as a holo-endemic area for Malaria [2-5] similar to coastal Tanzania where the transmission intensity (Entomologic Inoculation Rate) is estimated to range from 200 - 500 infective bites per year [4,11]. The observed parasite prevalence rates ranging from 30% to 50% are considerably lower than those previously published for Pemba Island in the late 1990's and place the island in a hyper-endemic, rather than a holo-endemic transmission category [12]. In spite of limited comparability of parasite prevalence rates in the age range 0-2 years [13], the difference is too large to be attributed to sampling variation over the age range. This observational cohort for malaria was nested in the larger vitamin A and zinc supplementation trials. This could also have a small impact on malaria rates observed here. Table 2 shows previously published figures on parasite prevalence for Pemba Island as well as some other sites in coastal East Africa. Other recent publications with sampling done around 2001-2003 confirm the lower transmission intensity observed in this study [14,15]. The highly successful control strategy of combining ACT with ITNs and IRS on the Zanzibar Archipelago [6] started after this study was carried out and further decreased parasite prevalence rates to almost zero in some districts of Zanzibar Island and to 1.4% for the most part of Pemba Island as of 2007 [16].

**Table 2:** Parasite prevalence rates from the literature on Pemba Island and coastal East Africa for the 1^st ^and 2^nd ^year of life

Parasite prevalence	Age group/N	Location/time of sampling	Source
**Pemba Island**			

76.90%	4-11 months	Kengeja village, Sept. 1996	Stoltzfus et al., J Nutr 130: 1724-33, 2000 [5]
			
	N = 39		

80.40%	12-23 months	Kengeja village, Sept. 1996	Stoltzfus et al., J Nutr 130: 1724-33, 2000 [5]
			
	N = 112		

33.90%	5-19 months	Wete district, Feb-May 2001	Olney et al., J Nutr. 137(12): 2756-62, 2007 [15]
			
	N = 771		

30%	5-9 months	Wete district, May-June 2002	Olney et al., J Nutr. 139(4): 763-772, 2009 [14]
			
	N = 256		

34%	14-Oct	Wete district, May-June 2002	Olney et al., J Nutr. 139(4): 763-772, 2009 [14]
			
	N = 315		

37%	15-19	Wete district, May-June 2002	Olney et al., J Nutr. 139(4): 763-772, 2009 [14]
			
	N = 270		

**Coastal East Africa**			

83.80%	6-40 months	Bagamoyo district,	Premji et al., Trop. Med. Parasitol. 46: 147-53, 1995 [11]
		
	N = 764	**Tanzania**	

42.50%	1-11 months	Kilifi south,	Snow et al., Lancet 349: 1650-54, 1997 [19]
		
	N = 511	**Kenya**	

7-12%	4-12 months	Manhica, **Mozambique**	Mayor et al., TMIH 8(1): 2-11, 2003 [20]
			
	N = 1875		

Regarding the successful malaria control intervention on the Zanzibar Archipelago, the estimate is questioned of the effect size that is implied when the baseline for the highly successful control strategy for Pemba Island is assumed to be that of a holo-endemic setting as the evidence suggests that transmission intensity was decreasing prior to the onset of the intensive malaria control programme. The lower parasite prevalence and incidence figures in our study could be caused by a number of reasons - some of which are directly related to the research study itself. The intensive follow-up including the provision of transport actually provided a better access to care. In addition, a quality control component for malaria diagnosis was implemented, which in itself could lead to lower estimates. Another contributing factor could be, by closely observing and treating malaria episodes as they occur in a cohort study, a situation similar to that of "intermittent presumptive treatment" was created, which as a control strategy was proven to decrease the number of malaria episodes in children [17]. As only a relatively small proportion of the total population (children up to two years of age) were treated with ACT, a mass effect of the new drug as observed on Zanzibar Island [6] is not probable as an underlying reason. Further underlying reasons could be present as the same trend of decreased malaria transmission is currently seen across a number of geographical locations in sub-Saharan Africa and yet not fully explained.

The micro-epidemiology on Pemba Island shows considerable heterogeneity of incidence and, therefore, presumably also heterogeneity in transmission. Figures were based on an area of approximately 20 square kilometres in the centre of Pemba Island (see Figure 4). Several countries have begun a serious move towards malaria elimination [18] - a goal that only recently became a realistic target. However, in order to plan realistically the relative impact of the tools used for control and elimination must be well understood in a variety of settings including the micro-epidemiological context.

Surveillance and micro-epidemiology of malaria has become increasingly important as pockets of malaria remain despite the intensive efforts and success of the malaria control programme [16]. The determinants of these pockets - from the molecular biology of the parasite to immunology of the human host need to be better understood.

Pemba Island was previously considered holo-endemic for malaria. The data collected in this study suggest that the transmission situation on Pemba Island was significantly lower in 2003-2005 suggesting a hyper-endemic or meso-endemic transmission environment. These figures were obtained just before the onset of the highly successful malaria control intervention by impregnated bed nets and IRS on the Zanzibar Archipelago and provide robust estimates of the malaria transmission situation prior to the control programme. Together with other published data, these results suggest that malaria transmission had started to decrease before the onset of the control programme. The local heterogeneity in malaria incidence highlights the importance of a micro-epidemiological approach in the context of malaria control and elimination.

## Competing interests

The authors declare that they have no competing interests.

## Authors' contributions

TJ, SS and DS conceived and designed the study. TJ, AD, SD, MR, MO and SS performed the study. TJ, RG, JT, SS and DS analyzed analysed the data. TJ and RG produced figures and tables. TJ, JT, SS and DS drafted the manuscript. All authors contributed to the writing of the manuscript and approved the final version.
